# Hepatoma-Derived Growth Factor: An Overview and Its Role as a Potential Therapeutic Target Molecule for Digestive Malignancies

**DOI:** 10.3390/ijms21124216

**Published:** 2020-06-13

**Authors:** Hirayuki Enomoto, Hideji Nakamura, Hiroki Nishikawa, Shuhei Nishiguchi, Hiroko Iijima

**Affiliations:** 1 Division of Gastroenterology and Hepatology, Department of Internal Medicine, Hyogo College of Medicine Nishinomiya, Hyogo 663-8501, Japan; nishikawa_6392_0207@yahoo.co.jp (H.N.); hiroko-i@hyo-med.ac.jp (H.I.); 2 Department of Gastroenterology and Hepatology, Nippon Life Hospital, Osaka 550-0006, Japan; nakamura.hideji@nissay-hp.or.jp; 3 Department of Internal Medicine, Kano General Hospital, Oska 531-0041, Japan; nishiguchi@heartfull.or.jp

**Keywords:** hepatoma-derived growth factor (HDGF), HDGF-related protein, nucleolin, cell growth, angiogenesis, apoptosis, digestive malignancies

## Abstract

Hepatoma-derived growth factor (HDGF) was identified in research seeking to find a novel growth factor for hepatoma cells. Subsequently, four HDGF-related proteins were identified, and these proteins are considered to be members of a new gene family. HDGF has a growth-stimulating role, an angiogenesis-inducing role, and a probable anti-apoptotic role. HDGF is ubiquitously expressed in non-cancerous tissues, and participates in organ development and in the healing of damaged tissues. In addition, the high expression of HDGF was reported to be closely associated with unfavorable clinical outcomes in several malignant diseases. Thus, HDGF is considered to contribute to the development and progression of malignant disease. We herein provide a brief overview of the factor and its functions in relation to benign and malignant cells. We also describe its possible role as a target molecule for digestive malignancies.

## 1. Introduction

A number of molecules have been proposed as potential targeted agents for malignant diseases [1]. One of our research aims was to determine a new molecule that could participate in the growth of hepatocellular carcinoma (HCC). As the Huh-7 cell line (a human HCC cell line) can proliferate in vitro, even under serum-free conditions, we explored its conditioned medium and identified a novel molecule, hepatoma-derived growth factor (HDGF) [2]. As expected, HDGF showed a growth-stimulating effect on hepatoma cells in vitro. In addition, studies with human HCC samples showed a higher expression of the HDGF protein in the cancerous tissues in comparison with the adjacent non-cancerous tissues [3]. Furthermore, the increased expression of HDGF was associated with invasive and aggressive phenotypes, and with poor clinical outcomes of several malignant diseases [4,5].

We previously reviewed the characteristics of the factor, and mentioned its possible relationship with the malignant features of HCC [3]. We herein provide a brief overview of the factor, including its functions in non-malignant cells, and then discuss its potential as a therapeutic target for digestive malignancies.

## 2. Overview of HDGF: Its Characteristics and Functions

### 2.1. HDGF and Its Related Proteins: Family Genes Characterized by Highly Homologous N-Terminal Regions

**Figure 1:**
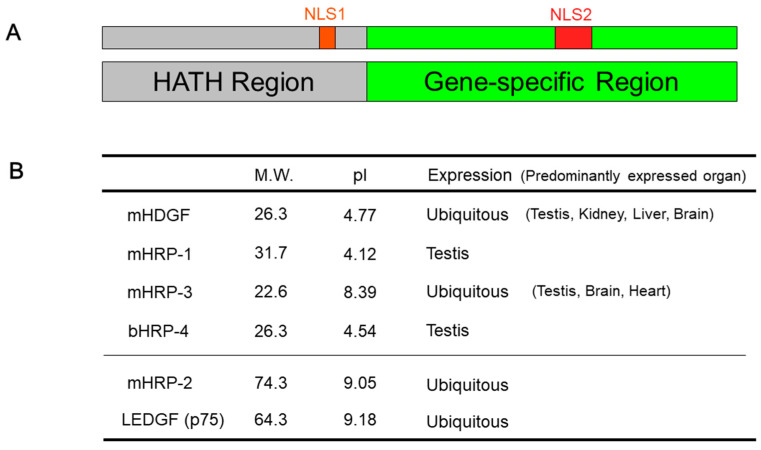
Basic structures and characteristics of hepatoma-derived growth factor (HDGF) and its related genes (**A**) The basic structure of hepatoma-derived growth factor (HDGF) and its related proteins [3]. The N-terminal region of the HDGF protein and those of HDGF-related proteins are highly homologous, and the conserved acid region with approximately 100 amino acids is named the HATH (homologous to the amino terminus of HDGF) region. HDGF and its related proteins contain two presumed nuclear localization signals (NLSs). These NLSs contain the basic amino acid-rich regions. The first NLS (NLS1) region and second NLS (NLS2) region reside in the HATH domain, and the gene-specific regions, respectively. (**B**) Characteristics of HDGF and HDGF-related proteins (HRPs). We reported the mouse sequences of HDGF (mHDGF), HRP-1 (mHRP-1), HRP-2 (mHRP-2) and HRP-3 (mHRP-3) [6,7]. The HRP-4 protein was originally determined as the bovine sequences (bHRP-4) [8]. The HDGF family proteins may be divided into the following sub-groups: the low molecular weight group (HDGF, HRP-1, HRP-3 and HRP-4) and the high molecular weight group (HRP-2 and LEDGF). M.W.; molecular weight, pI; isoelectric point.

**Figure 2:**
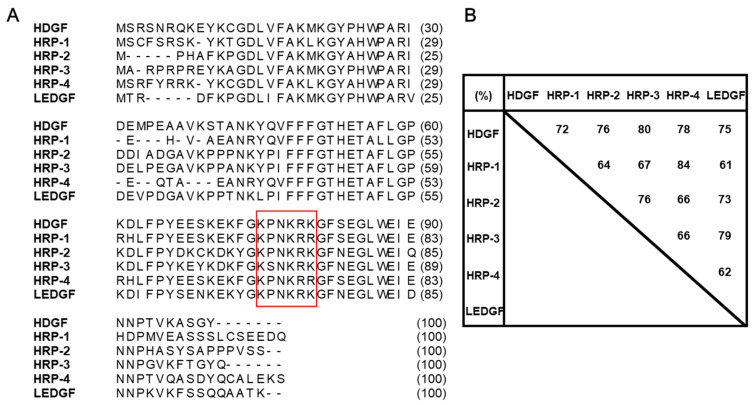
Homology of hepatoma-derived growth factor (HDGF) and HDGF-related proteins (HRPs) (**A**) Amino acid sequences of the HATH regions in the HDGF and its family proteins are shown. The numbers in parentheses indicate the amino acid number from the N-terminus of the proteins. (**B**) The homology of the HATH regions in the HDGF and its family proteins. High homology was observed [6,7,8,9]. The red square indicates the basic amino acid-rich regions (KPNKRK or KPNKRR; basic residues underlined) as the presumed first nuclear localization signal (NLS1) in the HATH regions. LEDGF; lens epithelium-derived growth factor.

HDGF is a heparin-binding glycoprotein that was identified in the mitogenic activity of Swiss 3T3 fibroblasts [2]. Subsequently, four new proteins have been also reported as HDGF-related proteins (HRPs), which include well-conserved N-terminal amino acid sequences (homologous to the amino terminus of HDGF; HATH region) [6,7,8]. In addition, Lens epithelium-derived growth factor (LEDGF) [9], a survival factor for the lens epithelium, also includes a HATH region. HDGF and its related proteins include two putative nuclear localization signals (NLSs) (Figure 1). The HRPs and LEDGF have highly homologous N-terminal regions, which contain the HATH domain (Figure 2), and which are considered to develop a new gene family.

### 2.2. HDGF as a Cellular Growth-Stimulating Factor

The HDGF protein was present in the conditioned media of cultured cells. However, the signal peptides for the secretion were not found in the sequence of the HDGF protein [2,3]. Nevertheless, several studies have shown that the exogenously administered HDGF protein stimulates the proliferation of several types of cells with the activation of growth signals, including the phosphorylation of mitogen-activated protein kinase (MAPK) signaling and/or hosphatidylinositol-3 kinases (PI3K) signaling [3]. Nüße et al. [10] reported two novel splicing variants of *HDGF* (*HDGF-B* and *HDGF-C*) that interacted with the components of the cytoskeleton and were functionally different from the major isoform of *HDGF* (*HDGF-A*). Recently, the possible exosomal secretion of HDGF was reported [11], and HDGF-A was shown to be located inside exosomes, whereas the new isoforms were located on the outer surface, suggesting that the role of the exosome-mediated pathway in new isoforms differed from that of HDGF-A [11]. Thus, the mechanisms underlying the secretion and receptor binding of HDGF and its related proteins require further study.

**Figure 3:**
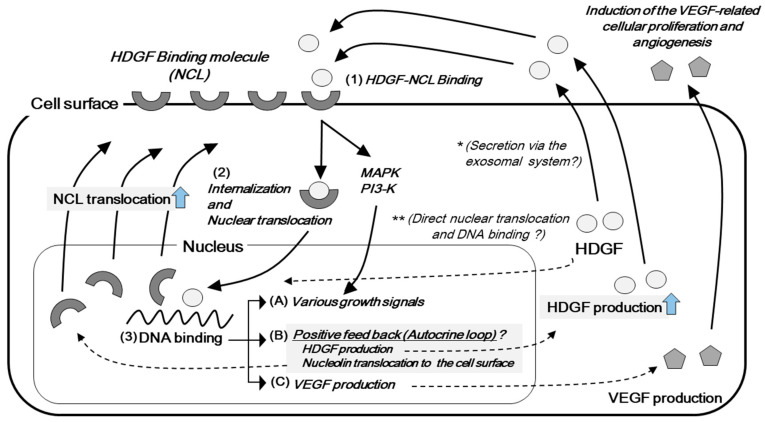
Schematic representation of the mechanisms of cellular proliferation by the hepatoma-derived growth factor (HDGF) (1) HDGF proteins bind to nucleolin (NCL) and (2) translocate to the nucleus [12]. (3) HDGF is suggested to bind to the genomic DNA. The homologous to the amino terminus of HDGF (HATH) region includes a proline–tryptophan-tryptophan-proline (PWWP) motif, which is suggested to bind to the promoter region of the target genes [12,13,14]. HDGF stimulates cellular proliferation via various pathways, including (**A**) the activation of intracellular signals, such as mitogen-activated protein kinase (MAPK) and/or the phosphatidylinositol-3 kinase (PI3K); (**B**) positive feedback, such as the promotion of HDGF production and mobilization of NCL to the cellular surface, and (**C**) the promotion of vascular endothelial growth factor (VEGF) production. The HDGF protein does not have peptides to signal the secretion of the classical Golgi system and may be secreted via the exsosomal system ^(^*^)^ [11]. However, the HDGF protein includes two putative nuclear localization signals (see Figure 1; Figure 2) and may function as a nuclear protein via direct translocation ^(^**^)^.

In 2015, Chen et al. showed that nucleolin (NCL) operates as a functional receptor of HDGF [12], and the presence of the receptor-mediated signal transduction pathways was confirmed (Figure 3). The binding of HDGF with NCL is suggested to translocate to the nucleus and promote cellular proliferation. The HATH region includes a proline-tryptophan-tryptophan-proline (PWWP) motif, and the motif is suggested to modify the transcription of the target genes through DNA-binding to their promoter regions [13,14]. The growth-stimulating effects of the HDGF protein are considered to have several mechanisms, including the stimulation of growth signals, such as MAPK and/or PI3K, the increased production of other growth factors, such as vascular endothelial growth factor (VEGF), and positive feedback of the HDGF/NCL axis, with the increased production of HDGF and NCL translocation to the cellular surface [12]. Lian et al. also reported the possible presence of a positive feedback loop in relation to the growth stimulation of HDGF [15]. However, despite the important finding of the HDGF/NCL axis, the HDGF protein includes two putative nuclear localization signals (Figure 1). As mentioned above, the secretion of a specific HDGF isoform was suggested [10], but its possible function as a nuclear protein via direct translocation to the nucleus cannot be eliminated [3,16,17].

### 2.3. HDGF as an Angiogenic Factor and a Possible Anti-Apoptotic Factor

Despite HDGF having been originally identified as having growth-stimulating activity, it was suggested to have various additional functions, including functions as an angiogenic factor and a possible anti-apoptotic factor [3]. 

HDGF was reported as a molecule that was possibly involved in the development of the cardiovascular system, and which was also involved in the healing of tissue damage [16]. HDGF was also shown to stimulate the proliferation of human umbilical vein endothelial cells, and to promote experimental tubular formation in vitro [18]. In addition, HDGF is considered to induce the production of the most major angiogenic factor, VEGF (see Figure 3). The transfection of the *HDGF* gene in NIH3T3 fibroblasts increased the expression of VEGF, and the transplanted HDGF-overexpressing NIH3T3 cells formed highly vascularized tumors in a xenograft model [18]. Additionally, HDGF-reduction in hepatoma cells resulted in the decreased expression of VEGF, and inhibited tumor growth in vivo [19]. Interestingly, the induction of VEGF by HDGF has also been shown in an oral cancer cell line [20]. Thus, HDGF is suggested to be involved in the progression of malignant diseases through two different mechanisms of angiogenesis: direct growth stimulation in endothelial cells, and the induction of VEGF. 

In addition to its role in angiogenesis, HDGF has been suggested to act as an anti-apoptotic factor. Although some studies have suggested the involvement of HDGF in the apoptotic pathway, an increasing number of studies have shown the possible participation of HDGF in resistance against apoptosis, rather than the induction of apoptosis [3,4]. In hepatoma cell lines, experimental studies demonstrated that the blocking of HDGF activated both extrinsic and intrinsic apoptotic pathways [21]. The inhibition of HDGF induced apoptosis in colorectal cancer cells [22]. Although these studies suggested that HDGF has an anti-apoptotic function, further studies are required to determine a conclusive role of HDGF in apoptosis.

### 2.4. Involvement of HDGF in Non-Transformed Hepatocyte Proliferation 

HDGF was identified as a growth-stimulating factor for HCC; however, we also found that HDGF was ubiquitously expressed in normal organs (Figure 1B) [2], suggesting some physiological role in non-cancerous tissues. We reported that HDGF was strongly expressed in fetal hepatocytes, particularly in the immature stage of the liver, and its expression was remarkably decreased near birth [23]. The expression of HDGF in hepatocytes decreases with cellular maturation, which suggests that the expression of HDGF is associated with the proliferative activity of hepatocytes. Furthermore, the administration of HDGF stimulates the proliferation of cultured fetal hepatocytes, whereas reducing the expression of HDGF severely suppresses the proliferation of these cells. These results strongly suggest a significant role of HDGF as a growth-stimulating factor for fetal hepatocytes during liver development [23]. 

In the normal state, the liver is a quiescent tissue, and most mature hepatocytes are out of the replicating phase. However, the liver has the capacity to regenerate in response to cell loss, such as after hepatectomy or drug-induced hepatic injury [24,25]. In both hepatectomized and CCl4-treated livers, the expression of HDGF was increased in hepatocytes, and the induction of HDGF was observed before the DNA synthesis peaked. The early induction of HDGF in parenchymal hepatocytes in the regenerating liver suggests the participation of HDGF in the proliferation of non-transformed adult hepatocytes [26]. These findings suggest that HDGF acts as a growth factor, not only in hepatoma cells, but also in proliferating non-transformed hepatic cells.

### 2.5. The Involvement of HDGF in the Proliferation of Various Types of Non-Transformed Cells

In the fetal stage, HDGF is highly expressed, not only in the liver, but also in various other tissues, including the kidneys, heart, lungs and gut. For some time after its discovery, HDGF was mainly discussed in relation to its role in non-transformed cells. Oliver et al. [27] reported that an endothelial growth factor, which they discovered in the conditioned media of a rat metanephrogenic mesenchymal cell line, was identical to HDGF. They mentioned that HDGF is likely to have an important function in glomerular capillary formation during renal development. As described above, HDGF is also abundantly expressed in fetal cardiovascular systems. The HDGF protein is first detected in atrial myocytes, then its expression expands to the ventricular myocytes, and endothelial and ventricular outflow cells. Additionally, HDGF is prominently expressed in the vascular smooth muscle cells (SMCs) and endothelial cells in the fetus, and the overexpression of HDGF promotes the growth of vascular SMCs [28]. In light of these findings, it is suggested that HDGF functions as a stimulating factor for vascular SMC proliferation, and contributes to the cardiovascular development and the repair of vascular injury. The expression of HDGF is also reported to be high in the endothelial cells of the blood vessels in the fetal lungs [29]. In a bleomycin-induced lung damage model, the expression of HDGF was found to be dominantly induced in bronchial and alveolar epithelial cells, such as type II alveolar cells [30], suggesting that HDGF is associated with organ development and the recovery of damaged tissues in the respiratory system. With regard to the proliferation of the gut system, the expression of HDGF is suggested to have a suppressive role in the maturation of fetal intestinal cells, and to be associated with the proliferation of these cells [31]. These historical early reports suggest that HDGF functions as a growth-stimulating factor for non-transformed cells, and is involved in the development of various organs and in the healing of tissue damage.

## 3. HDGF in Digestive System Malignancies

HDGF has been found to be involved in disease progression of several digestive malignancies, and the main findings in the previous reports were summarized in Table 1

### 3.1. HDGF in Hepatocellular Carcinoma

**Figure 4:**
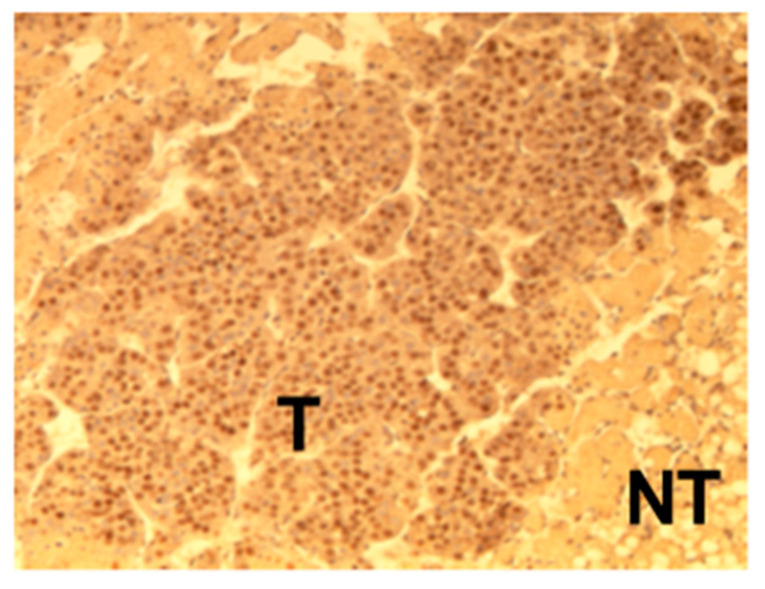
Immunostaining of HDGF in the liver tissue of the Fatty Liver Shionogi (FLS) mouse. HDGF was expressed more strongly in hepatocellular carcinoma tissues than in adjacent liver tissues. T: tumor tissue, NT: non-tumorous tissue.

As HDGF was originally identified as a growth factor for hepatoma cells, various studies have demonstrated that HDGF is associated with the progression of HCC [32,33,34,35,36,37]. The expression of HDGF was detected in several hepatoma-derived cell lines [2,3]. In addition, HDGF significantly increases the proliferation of hepatoma cells [16,36]. Moreover, HDGF was found to be associated with tumor growth in a xenograft model in vivo [19]. These in vitro and in vivo experimental studies strongly suggest that HDGF acts as a growth factor for hepatoma cells. We examined the expression of HDGF in the liver in two rodent HCC models. A choline-deficient amino acid (CDAA) diet causes steatohepatitis with progressive liver fibrosis in Fisher F344 rats. In this model, HCC was observed from after 52 weeks of age. The Fatty Liver Shionogi (FLS) mouse is a mouse strain that spontaneously develops a fatty liver. At 52 weeks, 90% of male FLS mice develop liver tumors that are pathologically confirmed to be hepatocellular adenoma and carcinoma [32]. The expression of HDGF in HCC tissues was higher than that in the adjacent cirrhotic liver tissues in CDAA-fed rats. Additionally, in FLS mice, HDGF was also expressed more strongly in HCC tissues, in comparison with the adjacent liver tissues with steatohepatitis (Figure 4). Of note, the expression of HDGF in the liver of FLS mice increased before the development of visible solid tumors, suggesting a growth-stimulating function of HDGF during the early stage of hepatocarcinogenesis, as well as during the progression of HCC [32].

In previous reports, the protein expression of HDGF in human HCC tissue samples was evaluated by immunostaining, and the HDGF protein was found to be more highly expressed in human HCC tissues than in the adjacent non-cancerous tissues [34,35]. Moreover, the expression of HDGF is strongly associated with the clinical course of HCC after surgery, and higher HDGF expression levels were found to be related to a poorer prognosis [34]. In fact, various independent groups have demonstrated that HCC patients with higher HDGF expression levels showed an unfavorable clinical outcome in comparison to those with lower HDGF expression levels [33,34,35,36,37], and the expression of HDGF was found to be independently associated with disease-free and overall survival after curative surgery in HCC patients. Accumulated findings suggest that HDGF has a significant clinical role in the disease progression of human HCC. Interestingly, a recent study suggested that HDGF was related to lipogenesis, and the change in the lipid metabolism might partly contribute to the mitosis of the hepatoma cells [37].

### 3.2. HDGF in Pancreatic Cancer

Pancreatic ductal carcinoma is known as one of the most aggressive malignant diseases, and is associated with a poor prognosis. HDGF is strongly expressed not only in hepatoma cells, but also in pancreatic cancer cells, including a number of pancreatic ductal carcinoma cell lines (i.e., MIA PaCa-2, PANC-1, PL45 and KP-4) [38]. We studied the HDGF expression in a total of 50 patients with primary ductal pancreatic carcinoma, and reported that the HDGF expression, as evaluated by immunostaining, could be an independent prognostic factor for pancreatic ductal carcinoma after curative resection [38]. Recently, HDGF was reported to be induced by transforming growth factor (TGF)-β1, and to contribute to the growth of pancreatic cancer cells through the anti-apoptotic effects on pancreatic stellate cells [39]. In addition, HDGF was suggested to be related to gemcitabine resistance in pancreatic cancer [40].

### 3.3. HDGF in Cholangiocarcinoma and Gallbladder Adenocarcinoma

Liu et al. [41] investigated the expression levels of HDGF and VEGF in patients with hilar cholangiocarcinoma, and showed that the high expression of HDGF was significantly associated with a poorer clinical outcome. Based on their multivariate analysis, they concluded that the expression of HDGF was an independent prognostic factor for hilar cholangiocarcinoma. HDGF has also been reported as an independent prognostic factor for extrahepatic cholangiocarcinoma [42]. In addition, the expression of HDGF is associated with the clinical biological behavior and prognosis of gallbladder adenocarcinoma [43]. Although the number of the studies was limited, these findings suggested that HDGF participates in the progression of biliary malignancies.

### 3.4. HDGF in Esophageal Cancer

In a previous study [44], a possible association between the expression of HDGF and the radiosensitivity of esophageal cancer cells was reported. HDGF was strongly expressed in radiation-sensitive esophageal cancer cells, and radiation therapy showed higher treatment efficacy in patients with a high expression of HDGF, in comparison to those with low HDGF expression levels [44]. Although the increased expression of HDGF led to higher treatment efficacy in patients who received radiotherapy, patients with higher HDGF expression levels showed unfavorable outcomes in comparison to those with lower expression levels, with lower disease-free and overall survival rates [45]. One possible explanation was that esophageal cancer cells with high HDGF expression levels have high proliferative activity, and thus finally cause an unfavorable outcome, regardless of the transiently observed high treatment efficacy. On the other hand, recent studies have suggested, based on in vivo experiments, that increased HDGF expression levels in irradiated fibroblasts may contribute to disease progression after radiation therapy [46]. Further studies are still necessary to determine the precise role of HDGF in relation to the poor prognosis in esophageal cancer.

### 3.5. HDGF in Gastric Cancer

HDGF is expressed in gastric cancer cells, and the reduction of HDGF in gastric cancer cells induced apoptotic signaling and reduced the invasive activity of the cells [47]. Interestingly, hepatocyte growth factor (HGF) induced HDGF in a dose-dependent manner, and HDGF induced the expression of VEGF, thus suggesting that HDGF may be involved in tumor growth by means of its cooperation with these growth factors [47]. In gastric cancer patients, higher HDGF expression levels are significantly associated with tumor infiltration, as well as vascular and lymphatic invasion [48]. Thus, HDGF is suggested to be significantly associated with the malignant characteristics of gastric cancer. Recently, increasing numbers of studies have also supported the possible involvement of HDGF in the disease progression of gastric cancer [49,50,51,52,53]. Further, the involvement of HDGF in *Helicobacter pylori*-related carcinogenesis has been suggested [50,51]. In addition, as suggested in hepatoma cells [37], the altered lipid metabolism may also be partially involved in the growth stimulation of gastric cancer cells caused by HDGF [53].

### 3.6. HDGF in Colorectal Cancer

The expression of HDGF is observed in human colorectal cancers [31], and HDGF has been shown to stimulate the proliferation of colorectal cancer cells [22]. We have documented that recombinant HDGF stimulated the proliferation of colonic HT-29 cells, whereas a polyclonal antibody against recombinant HDGF significantly suppressed their proliferation [54]. The knockdown of HDGF was reported to activate the mitochondrial apoptotic pathway [22], and a recent study revealed the involvement of HDGF in the proliferation and invasion of colorectal cancer cells [55]. In light of these findings, HDGF is considered to play an important role in gut epithelial cell proliferation, including the proliferation of colorectal cancer cells.

### 3.7. HDGF in Gastrointestinal Stromal Tumors (GIST)

The HDGF protein was detected in GIST tissues [56,57]. An immunohistochemical evaluation suggested that there is a significant relationship between the expression of HDGF and tumor growth. The expression of HDGF is correlated with tumor mitosis and tumor size. High HDGF expression levels in patients with GIST were related to early recurrence and a poor prognosis, and the expression of HDGF was reported to be independently associated with the disease-free and overall survival of patients after surgical resection [56]. Furthermore, with regard to surgically resected colorectal stromal tumors, HDGF was reported to be an independent prognostic factor in patients with colorectal stromal tumors, since patients with high HDGF expression levels showed early recurrence and a poor prognosis [57].

## 4. HDGF as a Potential Target Molecule for Anticancer Therapy

As described above, HDGF participates in the progression of various digestive malignancies, and serves as an independent prognostic factor [3,4,5]. Our experimental studies showed that anti-HDGF therapy significantly suppressed the growth of cancer cells, such as hepatoma cells and colonic cancer cells [16,19,54]. Since clinical studies showed that high HDGF expression levels were associated with a poor prognosis in several malignant diseases, we consider that the inhibition of HDGF could provide a new strategy for cancer therapy. Importantly, as described above, HDGF-related proteins form a gene family. It is well known that family genes sometimes share redundant functions in order to compensate their roles, and the inactivation of one gene minimally affects physiological conditions [58,59]. Indeed, *HDGF*-deficient mice did not show any notable phenotype [60]. Since reversing the overexpression of HDGF inhibited tumor growth in vivo [19,55], the reduction of HDGF could be expected to suppress the proliferation of malignant cells, with nominal influence on non-cancerous tissues. The fact that HDGF is a representative member of a gene family ( Figure 1; Figure 2) might be advantageous when we consider it as a therapeutic target. Recently, the significant roles of HDGF in malignant diseases were reported for many non-digestive malignant diseases, including osteosarcoma [61,62], endometrial cancer [63,64], lung cancer [65,66,67], malignant glioma [68,69,70], cervical cancer [71], ovarian cancer [72] and breast cancer [73]. Thus, HDGF is considered as a potential target molecule for anticancer therapy.

HDGF expression is highly observed in proliferating cells, such as those in developing organs, healing tissues and malignant tissues. Zhao et al. [74] suggested that HDGF might be a target for repressing cancer stem cell proliferation and preventing the recurrence of lung cancer after chemotherapy. They also showed that anti-HDGF treatment reduced the expression of Notch protein. In addition, the Wnt pathway genes Wnt1 and Frizzled were severely suppressed, with 8- and 51-fold reductions, respectively. These findings suggested that HDGF may be a target of anticancer therapy, as a factor involved in the stemness of cancer cells.

## 5. Future Perspectives

Although HDGF is considered as a potential target for novel cancer therapies, it remains unclear how HDGF and its signal(s) can be targeted to provide reliable anticancer therapy. Although HDGF may be negatively regulated by vitamin K2 [75,76], no available drug that effectively targets the HDGF molecule has been identified. Recently, non-coding RNAs, such as microRNAs and long non-coding RNAs (lncRNAs), which modulate the function of the target genes, have been shown to have an important role in several diseases [77,78]. Indeed, various microRNAs [40,61,62,64,65,66,67,69,72] and lncRNAs [49,61,69,71,73] have been suggested to be involved in the regulation of the functions of HDGF, findings that may support the development of HDGF-targeting therapies. For instance, lncRNA HLA complex P5 (lnc HCP5) was reported to cause gemcitabine resistance in pancreatic cancer cells, through the upregulation of miR-214-3p and its target gene, HDGF [40]. In addition, lncRNA testis development-related gene 1 (TDRG1) was shown to interact with miR-873-5p and increase the expression of HDGF in gastric cancer cells [49]. However, these newly proposed mechanisms differed among the studies, and the results appear to depend on the cell lines that were used. Thus, the regulation of HDGF still needs to be further studied in order to establish viable anticancer strategies.

## 6. Conclusions

HDGF was identified in order to find a novel growth-stimulating factor for hepatoma cells. HDGF and HRPs were determined as members of a new gene family. HDGF is involved in various events in which it exerts its multiple functions, including cellular proliferation, angiogenesis and anti-apoptosis. HDGF is suggested to contribute to the proliferation of non-cancerous cells, and promote organ development and tissue repair. An increasing number of studies have shown that the overexpression of HDGF is correlated with unfavorable clinical outcomes in several malignant diseases, suggesting that HDGF could be a new molecular target for cancer therapy. Clarifying the regulation of HDGF will lead to novel treatment strategies for malignant diseases.

## Figures and Tables

**Table 1:** Digestive malignancies in which HDGF has been found to be involved in disease progression.

Malignant Digestive System Diseases	Main Findings	Reference(s)
Hepatocellular carcinoma	HDGF acts as a growth factor for hepatoma cells in vitro and in vivo. The HDGF protein was found to be more highly expressed in human HCC tissues than in the adjacent non-tumorous tissues. The HDGF expression level is strongly associated with the clinical course of HCC, and the higher expression of HDGF was found to be related to a poorer prognosis. HDGF is suggested to be related to lipogenesis, and the change in the lipid metabolism may contribute to the mitosis of hepatoma cells.	[2,3,16,19,32,33,34,35,36,37]
Pancreatic cancer	The immunohistochemically determined HDGF expression was found to be an independent prognostic factor for pancreatic ductal carcinoma after curative resection. HDGF may contribute to the growth of pancreatic cancer cells through the anti-apoptotic effects on pancreatic stellate cells.	[38,39,40]
Cholangiocarcinoma	High HDGF expression levels were significantly associated with poorer overall survival in patients with hilar cholangiocarcinoma. The expression of HDGF was also reported as an independent prognostic factor for extrahepatic cholangiocarcinoma.	[41,42]
Gallbladder adenocarcinoma	The expression of HDGF is associated with malignant biological behavior and an unfavorable prognosis of gallbladder adenocarcinoma	[43]
Esophageal cancer	Patients with high HDGF expression levels showed high sensitivity to the radiotherapy. However, the higher expression of HDGF led to a poor outcome. Recently, the increased expression of HDGF in irradiated fibroblasts was suggested to promote the malignant tendency of esophageal cancer cells.	[44,45,46]
Gastric cancer	HDGF stimulated cellular proliferation and promoted the growth of gastric cancer cells. HDGF was suggested to induce VEGF. The possible involvement of HDGF in the disease progression of gastric cancer was suggested. Altered lipid metabolism may be partly involved in the stimulation of gastric cancer cell growth by HDGF.	[47,48,49,50,51,52,53]
Colorectal cancer	The expression of HDGF is remarkably high in human colorectal cancers. HDGF is suggested to promote the proliferation and invasion of colorectal cancer cells.	[22,31,54,55]
Gastrointestinal stromal tumor	The high expression of HDGF in patients with GIST was related to early recurrence and a poor prognosis. The expression of HDGF was shown to be an independent prognostic factor for the clinical outcome of patients after surgical resection of GIST.	[56,57]

HDGF, hepatoma-derived growth factor; VEGF, vascular endothelial growth factor; GIST, gastrointestinal stromal tumor. Overall reviews [3,4,5] and references therein should be beneficial.

## Abbreviations

HCC	Hepatocellular carcinoma
HDGF	Hepatoma-derived growth factor
HRP	HDGF-related protein
HATH	Homologous to the amino terminus of HDGF
LEDGF	Lens epithelium-derived growth factor
NLS	Nuclear localization signal
MAPK	Mitogen-activated protein kinase
PI3K	Phosphatidylinositol-3 kinase
NCL	Nucleolin
PWWP	Proline-tryptophan-tryptophan-proline
VEGF	Vascular endothelial growth factor
SMC	Smooth muscle cell
CDAA	Choline-deficient amino acid
FLS	Fatty Liver Shionogi
GIST	Gastrointestinal stromal tumor
LncRNA	Long non-coding RNA
